# Characterization of biofilm formation and virulence factors of *Staphylococcus aureus* isolates from paediatric patients in Tehran, Iran

**DOI:** 10.22038/ijbms.2020.36299.8644

**Published:** 2020-05

**Authors:** Hiva Kadkhoda, Zohreh Ghalavand, Bahram Nikmanesh, Mansoor Kodori, Hamidreza Houri, Donya Taghizadeh Maleki, Ali Karimi Bavandpour, Gita Eslami

**Affiliations:** 1 Department of Microbiology, School of Medicine, Shahid Beheshti University of Medical Sciences, Tehran, Iran; 2 Department of Medical Laboratory Sciences, School of Allied Medical Sciences, Tehran University of Medical Sciences, Tehran, Iran; 3 Department of Bacteriology, Faculty of Medical Science, Tarbiat Modares University, Tehran, Iran

**Keywords:** Biofilm, Fibronectin binding-proteins, MRSA, MSCRAMMs, Pediatric, Staphylococcus aureus, Virulence factors

## Abstract

**Objective(s):**

*Staphylococcus aureus* can cause several infections. Its capability to form biofilm has been reported to be a vital property involved in the bacteria’s pathogenesis. Various genes contributing to biofilm formation have not yet been completely clarified. This study was designed to evaluate the factors influencing adherence and biofilm formation in *S. aureus* isolated from paediatric patients.

**Materials and Methods:**

One hundred and ninety-seven *S. aureus* isolates were obtained from pediatric patients and confirmed with phenotypic and molecular examinations. Antimicrobial susceptibility testing and biofilm formation were evaluated using standard methods. The genes encoding adhesion and virulence factors were investigated by the PCR method.

**Results:**

The most efficient antibiotics against *S. aureus* isolates were vancomycin and linezolid. Approximately, 54.2% of MSSA and 85.6% of MRSA isolates were biofilm producers according to the microtiter test. Our analysis indicated that MRSA isolates are better able to form biofilm compared with MSSA isolates. All isolates harbored *clfA, fnbpA, icaA, icaB, icaC*, and *icaD*, while *clfB, fnbB, hlg*, and *pvl* were detected in 99.5%, 42.1%, 97.5%, and 5.6% of isolates, respectively. In addition, a significant difference was found in *fnhB* gene and biofilm formation.

**Conclusion:**

Our findings showed a significant correlation between *mecA* and *pvl* genes and MRSA and biofilm formation in *S. aureus* isolates. Additionally, this study indicated the significant role of the *fnhB* gene as a major marker for S. aureus biofilm formation. Therefore, further experiments are warranted to exactly elucidate the function of the *fnhB* gene in the formation of biofilm.

## Introduction

*Staphylococcus aureus *has long been considered one of the main human pathogens that is involved in the induction of a series of clinical infections. *S. aureus *is one of the most prevalent causes of skin and soft tissue infections, infective endocarditis, nosocomial infections, including various surgical wound infections as well as bacteremia (1, 2). The most vulnerable group to the serious complications of *S. aureus* infections are infants and children, who could be affected with this bacteria either in the community or in the hospital (3). With expressing a group of different virulence factors that remarkably participate in the host–pathogen interactions, pathogenic *S. aureus* strains colonize and induce infection. These virulence factors not only facilitate the entrance of the pathogen into host tissues, immune response evasion, and adherence to the host cells, but also induce tissue damage by secreting exoenzymes and toxins (4, 5). 

*S. aureus* strains also produce a multi-layered biofilm embedded within the slime layer on which a heterogeneous protein is expressed. The significant characteristic of the biofilms is their innate resistance to host immune defenses and antimicrobial agents, which lead to chronic and destructive infections (6). The biofilm formation allows *S. aureus *to attach and persist on native host tissues, such as heart valves and bone to cause infective endocarditis and osteomyelitis, respectively, or on implanted medical devices, including catheters, prosthetic joints, artificial heart valves, and orthopedic implants to develop severe chronic infections in hospitalized patients in healthcare environment (7, 8). The first step in the formation of biofilm by *S. aureus* is adherence to various surfaces and colonization of host tissues. For this purpose, the bacterium expresses several surface adhesions named microbial surface components, recognizing adhesive matrix molecules (MSCRAMMs) such as fibronectin-binding proteins A and B (*finbA* and *finbB*), clumping factors A and B (*clfA* and *clfB*), collagen-binding protein (*cna*), bone sialoprotein binding protein (*bbp*), and fibrinogen binding protein (*fib*) (9, 10). The next step is the expression of *icaADBC* operon and a surface protein, known as biofilm-associated protein (*bap*). *icaADBC *operon is accountable for synthesis of the extracellular polymeric substances of *s. aureus* biofilms, polysaccharide intercellular adhesin (PIA), which mediates bacterial cell to cell adhesion and biofilm formation. Among the *ica* genes, *icaA* and *icaD* have shown a major function in the biofilm formation in *S. aureus* strains (11, 12).

In addition to the high pathogenicity for humans, *S. aureus* is notorious for developing a resistance phenotype to antimicrobial agents. Methicillin-resistant *S. aureus* (MRSA) isolates have been introduced as major causes of nosocomial- and community-acquired- *staphylococcal* infections, which widely outbreak throughout the world and cause serious problems in infection prevention and control in health care facilities. (13, 14). 

The participation of biofilms formation in clinical isolates of *S. aureus* has received increasing attention, since there is a correlation between biofilms, chronic infections, and resistance to antimicrobial agents. Accordingly, it is still essential to extend the research the genes modulating biofilm formation, antibiotics resistance, and association with *s. aurous* infections. Therefore, this study was conducted to characterize the virulence factors and biofilm formation in two groups of MRSA and MSSA of *S. aureus* isolates from pediatric patients in Tehran.

## Materials and Methods

***The isolation and the identification of S. aureus***

For the present study 197 *S. aureus *isolates were collected from children with clinical symptoms of infection, including leucocytosis and fevers admitted to Children’s Medical Center Hospital, from January 2016 to September 2017 in Tehran. Only one isolate from each patient was used. We also applied both morphological and biochemical analyses such as Gram staining, catalase, haemolysis, oxidase, coagulase, DNase, and mannitol fermentation tests on the colonies collected from the primary cultures. In addition, to confirm the presence of *S. aureus *in the isolates, the expression of the *nuc* gene was studied using PCR analysis (Table 1 summarizes the list of the used primers) (15). For more analysis, the isolates were maintained at -70 ^°^C in a medium of trypticase soy broth (TSB) (Merck Co, Germany) comprising 10% glycerol. The study procedures were conducted under the permission of the ethics committee of Shahid Beheshti University of Medical Sciences (IR.SBMU.MSP.REC.1395.199).

***Antimicrobial susceptibility testing***

To test the antibiotic susceptibility, we used the standardized Kirby-Bauer disc-diffusion method on Mueller-Hinton agar (Merck co., Germany). To test this susceptibility, we have chosen those antimicrobial disks that are commonly used in the treatment protocol of *S. aureus* infections according to the Clinical and Laboratory Standards Institute (CLSI, 2017). Those antibiotics were as follows: penicillin (10 units/disk), trimethoprim/sulfamethoxazole (1.25/23.75 μg/disk), chloramphenicol (30 mg), ciprofloxacin (5 mg), clindamycin (2 mg), gentamicin (10 μg/disk), kanamycin (30 mg), minocycline (30 μg/disk), erythromycin (15 μg/disk), rifampicin (5 μg/disk), and linezolid (30 μg/disk). By using E-test (Liofilchem Co, Roseto, Italy) and through evaluating the minimum inhibitory concentration (MIC), the extent of vancomycin susceptibility was examined. It is worth noting that for quality control reference *S. aureus* ATCC 25923 was used. Herein, a multi-drug resistant (MDR) *S. aureus* strain was determined to be a single isolate that displayed resistance to three or more unique antimicrobial classes.

***Detection of MRSA strains***

MRSA strain phenotypic detection was conducted by using the disk diffusion method on the basis of CLSI guidelines. Those isolates which had an inhibition zone size equal to or less than 19 mm were claimed as MRSA strains. To more precisely confirm the MRSA isolates, we also checked *mecA* amplification (Table 1).

***Biofilm formation assays***

*Congo red agar method*

For evaluating the capability of *S. aureus* to form biofilm, the isolates were cultivated on brain heart infusion agar (Merck Co, Germany) with 0.08% (w/v) Congo red (Sigma-Aldrich Co, Germany) supplemented with 3.6% (w/v) sucrose. The inoculated plates were subsequently kept at 35 ^°^C under aerobic conditions. According to the method described by Freeman *et al. *those organisms which form black colonies with a dry crystalline consistency are strong-biofilm producer strains and those producing black colonies with the absence of a dry crystalline are categorized as a moderate-biofilm producers (20). Non-biofilm producer strains remained smooth red colonies, with occasional darkening at the centers of colonies. For positive and negative control, we have chosen two reference strains, ATCC 25923 and ATCC 12228, respectively.

***Colorimetric microtiter plate (MTP) assay***

The ability to adhere to a polystyrene microtiter plate was used to assess the extent of biofilm production (21). The bacterial suspension from an overnight blood agar was provided with a 0.5 McFarland standard with sterile TSB (Merck Co., Germany). For evaluating the biofilm, we added 300 μl of a provided bacterial suspension to 3 wells of a 96-well microtiter plate and filled the other 3 wells with uninoculated sterile TSB medium as negative control. After 4 hr and discarding the supernatant, each well was washed with 300 μl phosphate-buffered saline (PBS), and then 300 μl of fresh TSB medium was added to each well. After 24 hr, the process of discarding the supernatant and washing the wells with PBS were repeated. Then for fixation, we incubated the wells for a further 15 min with 300 μl methanol with the purity of 99%. Wells were then stained with crystal violet (0.1% w/v). The amount of staining was examined at the wave length of 590 nm using a spectrophotometer. All steps were performed at room temperature and repeated three times. Based on the calculation method described by Stepanovic *et al.*, a cut-off optical density (ODc) was defined as three standard deviations (SD) above the mean optical density (OD) of the negative control (uninoculated medium): ODc=average OD of negative control+(3×SD of negative control) (22). ODc value was calculated for each microtiter plate separately. For interpretation of the results, strains were divided into the following categories: OD ≤ODc=non-biofilm producers; ODc <OD ≤2×ODc=weak biofilm producers; 2×ODc <OD ≤4×ODc=moderate biofilm-producers; and 4×ODc <OD=strong biofilm producers.

***Detection of biofilm-associated and virulence genes by PCR reaction***

For extracting the genomic DNA, we used High Pure PCR Template Preparation Kit (Roche Co., Germany). Eluted DNA was kept at -20 ^°^C for later analysis. The presence of the biofilm-associated and virulence genes including *clfA*, *clfB*, *finbA*, *finbB*, *bap*, *icaA*, *icaB*, *icaC*, *icaD*, *pvl*, and *hlg* were examined via separate standard PCR reactions. List of the used primers are summarized in Table 1. PCR assay was performed in an ultimate volume of 25 μl of 1X PCR reaction buffer, 10 pmol primer, 1 μl DNA template (3 μg/μl), 1.5 mM MgCl2, 0.2 mM dNTP mixture, and 5 U of *Taq* DNA polymerase (Cinnaclon, Tehran, Iran). The temperature protocol for PCR analysis was as follows: an initial denaturation step at 94 ^°^C for 5 min, denaturation step at 94 ^°^C for 2 min, annealing step for 1 min, and final extension step at 72 ^°^C for 5 min. The quality of the PCR products were evaluated using electrophoresis at 120 V for 45 min. 

***Statistical analyses***

Chi-square test and independent *t*-test were used to compare the categorical variables and to evaluate the differences in means, respectively. The *P*-values less than 0.05 were considered statistically significant.

## Results

***Demographic data and bacterial detection***

**Figure 1:**
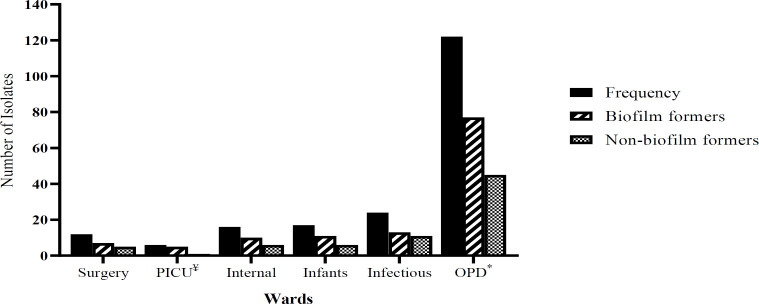
Characterization of biofilm formation among *S. aureus* isolates according to the wards of hospital

**Figure 2:**
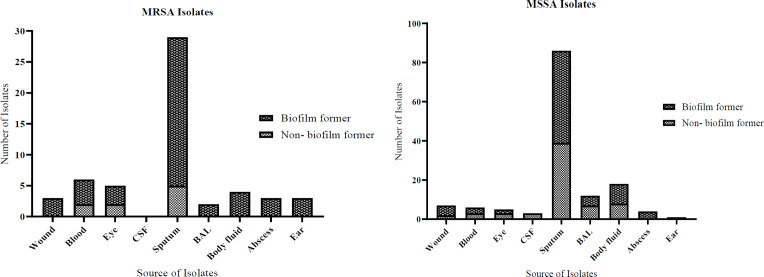
Biofilm formation in MRSA and MSSA strains from paediatric patients according to origin of sample

MRSA: Methicillin-resistant *staphylococcus aureus; *MSSA: Methicillin-susceptible *staphylococcus aureus; *CSF: Cerebrospinal fluid; BAL: Bronchoalveolar lavage

In this study, 197 clinical isolates of *S. aureus* were obtained from children under 15 years of age in a 20 month time period. Among them, 122 isolates (61.9%) were gathered from outpatient (OPD) or emergency department, including infection 24 (12.2%), infants 17 (8.6%), internal 16 (8.1%), surgical 12 (6.3%), and pediatric intensive care unit (PICU) 6 (3%) (Figure 1). In total, 115 (58.4%) of *S. aureus* isolates were collected from sputum, and the residual 42 isolates were harvested from body fluid 22 (11.2%), bronchoalveolar lavage (BAL) 14 (7.1%), blood 12 (6.1%), wound 10 (5.1%), eye discharges 10 (5.1%), abscess 7 (3.6%), ear discharges 4 (2%), and cerebrospinal fluid (CSF) 3 (1.6%) (Figure 2). 

***Antibiotic resistance profiles***

The results obtained from the antibiotics susceptibility test revealed that linezolid and vancomycin (100% as the susceptibility rate) were the most potent antibiotics against isolates. The susceptibility rate of other antibiotics are as follows: minocycline 192 (97.5%), rifampicin 183 (92.9%), sulfamethoxazole/trimethoprim 179 (90.9%), kanamycin 173 (87.8%), and gentamicin 172 (87.3%). The extent of resistance to erythromycin, ciprofloxacin, clindamycin, and chloramphenicol was assessed as 92 (46.7%), 86 (43.7% (, 82 (41.6%), and 79 (40.1%) ,respectively. 192 (97.5%) of isolates displayed a resistance phenotype to penicillin. The MIC50 and MIC90 for vancomycin varied from 0.5 to 4 µg/ml for MRSA (1 to 4 µg/ml) and MSSA (0.5 to 2 µg/ml) isolates. We found that 32% of isolates could be regarded as MDR *S. aureus* strains. In addition, among the isolated *S. aureus*, 55 (27.9%) were detected as MRSA strains by phenotypic and molecular approaches.

***Biofilm formation***

To examine the ability to generate biofilm, both methicillin-susceptible *S. aureus* (MSSA) and MRSA isolates were used. The results of Congo red agar showed that from the total number of *S. aureus* isolates, 71 (36%) produced red colonies (non-biofilm producers), 115 (58.4%) formed almost black colonies without a dry crystalline consistency (moderate- biofilm producers), and 11 (5.6%) produced very black colonies with a dry crystalline consistency (strong-biofilm producers) (Table 2). Biofilm development assessed by microtiter plate method indicated that out of 142 MSSA and 55 MRSA isolates, 77 (54.2%) and 46 (83.6%) isolates were able to adhere to polystyrene microtiter plate and produce biofilm, respectively. Of these, 3 (2.1%) and 12 (8.5%) of MSSA and 4 (7.3) and 9 (76.4) of MRSA isolates exhibited strong and moderate biofilm production, respectively. In contrast, 62 (43.7%) and 65 (45.8%) of MSSA and 33 (60%) and 9 (16.4%) of MRSA exhibited low level and non-biofilm formation, respectively. The results of MTP and Congo red methods showed that the ability of MRSA isolates to generate biofilm is more potent than MSSA isolates, suggesting that probably there is a significant association between the resistance to oxacillin and biofilm formation (*P*-value≤0.05). Comparison between antibiotics resistance and biofilm formation does not show a correlation in MRSA isolates but in MSSA isolates a correlation was identified in resistance to gentamycin, kanamycin, and chloramphenicol biofilm formation (*P*-value ≤ 0.05) (supplementary material Tables S1, S2). The results do not show any correlation between type of specimen, source of isolate, and biofilm formation among *S. aureus* isolates (Figures 1 and 2). 

***Presence of biofilm-associated and virulence genes***

The results obtained from molecular analysis of different biofilm-associated and virulence determinants revealed that all isolates harboured *clfA*, *fnbpA,* *icaA*, *icaB*, *icaC*, and *icaD*, while *clfB*, *fnbB*, *hlg*, and *pvl* were detected in 99.5%, 42.1%, 97.5%, and 5.6% of isolates, respectively. In MSSA and MRSA isolates the *fnbB* gene were detected in 38 (69.09%) and 45 (31.7%), respectively, so a significant difference was seen in *fnbB* gene distribution and biofilm formation in two groups (*P*-value ≤ 0.05). Moreover, the comparison between the biofilm-producing and non-biofilm producing isolates indicated that there was no obvious correlation between gene and the production of biofilm. In addition, we found that unlike *fnbB *presence, which differs between MSSA and MRSA isolates, the presence of other genes has no association with biofilm formation. In our study, no strain harboured the *bap* gene. Additionally, we found a remarkable association between the existence of *mecA* and *pvl* genes (*P*-value= 0.012). Table 3 describes that there is a link between the generation of biofilm and the presence of MSCRAMM and *icaADBC* genes in MSSA and MRSA isolates. For each gene, a sample was randomly sequenced and the related data are available on NCBI (supplementary material, Table S3).

**Table 1.** List of the oligonucleotide primers

**Gene target**	**Sequences (5’-3’)**	**Product size (bp)**	**Annealing temperature (C°)**	**Reference**
*Nuc*	F: GCGATTGATGGTGATACGGTT R: AGCCAAGCCTTGACGAACTAAAGC	270	54	(16)
*MecA*	F: GTAGAAATGACTGAACGTCCGATAA R: CCAATTCCACATTGTTTCGCTCTAA	310	55	(17)
*ClfA*	F: ATTGGCGTGGCTTCAGTGCT R: CGTTTCTTCCGTAGTTGCATTTG	280	54	Designed in this study.
*ClfB*	F: GCAGCATTTACTACCGGTTC R: CTACAACAGAGCCAGCTTCA	301	55	Designed in this study.
*FinA*	F: CACTGCGCCAGTTACAATAC R: GATGGTGGAGGTGGATATGT	306	55	Designed in this study.
*FinB*	F: TCTCTGCAACTGCTGTAACG R: GGAAAGTGGGAGTTCAGCTA	320	55	Designed in this study.
*Bap*	F: CCCTATATCGAAGGTGTAGAATTG R: GCTGTTGAAGTTAATACTGTACCTGC	971	52	(18)
*IcaA*	F: GGAGGTCTTTGGAAGCAAC R: TGCGACAAGAACTACTGCTG	390	55	Designed in this study.
*IcaB*	F: TTGCCTGTAAGCACACTGGA R: GGAGTTCGGAGTGACTGCTT	735	55	Designed in this study.
*IcaC*	F: GAACAACACAGCGTTTCACGA R: TGCGTGCAAATACCCAAGAT	294	55	Designed in this study.
*IcaD*	F: GCCCAGACAGAGGGAATAC R: CGCGAAAATGCCCATAGT	229	54	Designed in this study.
*Hlg*	F: GCCAATCCGTTATTAGAAAATGC R: CCATAGACGTAGCAACGGAT	937	55	Designed in this study.
*Pvl*	F: CTCTAGCCGATGTCGCTCAA R: ATACCTGAGGCTCGCCACTG	433	55	(19)

PICU: Pediatric intensive care unit, OPD: Outpatient department

**Table 2.** The formation of biofilm by microtiter plate and Congo red agar methods in isolates of *staphylococcus aureus*

**Method**	**Biofilm production**	**MSSA (n = 192)** **N (%)**	**MRSA (n = 55)** **N (%)**	**Total** **N (%)**
Congo red agar	Very black	8 (5.6)	3 (5.5)	11 (5.6)
	Black	21 (14.8)	8 (14.5)	29 (14.7)
	Weak	53 (37.3)	33 (60.0)	86 (43.7)
	Red	60 (42.3)	11 (20.0)	71 (36.0)
Microtiter plate	Strong	3 (2.1)	4 (7.3)	7 (3.6)
	Moderate	12 (8.5)	9 (16.4)	21 (10.7)
	Weak	62 (43.7)	33 (60.0)	95 (48.2)
	None	65 (45.8)	9 (16.4)	74 (37.6)

MRSA: Methicillin-resistant *staphylococcus aureus; *MSSA: Methicillin-susceptible *staphylococcus aureus*

**Table 3:** The relationship between biofilm generation and MSCRAMM and *icaADBC* genes in MSSA and MRSA isolates from pediatric patients

**Virulence determinants gene**		**MRSA (n= 55)**		**MSSA (n= 142)**	
		**Biofilm formation (OD590 nm)**		**Biofilm formation (OD590 nm)**	
		**None**	**Positive**	**None**	**Positive**
***Bap***	Negative	9	46	65	77
	positive	0	0	0	0
***ClfA***	Positive	9	46	65	77
	Negative	0	0	0	0
***ClfB***	Positive	9	45	65	77
	Negative	0	1	0	0
***fnhpA***	Positive	9	46	65	77
	Negative	0	0	0	0
***fnhpB***	Positive	3	35	2	43
	Negative	6	11	63	34
***Hlg***	Positive	9	43	65	75
	Negative	0	3	0	2
***Pvl***	Positive	2	5	1	3
	Negative	7	41	64	74
***IcaA***	Positive	9	46	65	77
	Negative	0	0	0	0
***IcaB***	Positive	9	46	65	77
	Negative	0	0	0	0
***IcaC***	Positive	9	46	65	77
	Negative	0	0	0	0
***IcaD***	Positive	9	46	65	77
	Negative	0	0	0	0

MRSA: Methicillin-resistant *staphylococcus aureus; *MSSA: Methicillin-susceptible *staphylococcus aureus*

## Discussion

The formation of biofilm in *S. aureus* is a critical step in the development of chronic infections. Accordingly, in order to control and manage these infections understanding the biofilm formation mechanism is thought to be important (6). Moreover, apparently, the capability of bacteria to generate biofilm is a characteristic that possesses tight correlation with the development of resistance to the antimicrobial agents, as it seems that many resistant species of bacteria are able to form multilayer biofilms (23, 24). In the current study, we evaluated phenotypic and genotypic characteristics of biofilm formation among clinical isolates of *S. aureus*, which may affect the chronicity and drug resistance in pediatric infections. The phenotypic and genotypic assays revealed a relatively low prevalence of MRSA in this study and this finding is in accordance with the recent report by Benvidi *et al.* among pediatric patients (25). However, our results contrast with the higher prevalence of MRSA infections previously reported in Iran (26, 27). These dissimilarities may be due to the effectiveness of screening methods for MRSA detection. In addition, the discrepancy between our results and the previous experiments could be a result of the sample population, as the other studies have included all age groups and healthy carriers, and our study was only carried out on pediatric samples.

Similar to other parts of the world, antibiotic resistance in clinical isolates of *S. aureus is a* severe, as well as challenging, public health problem in Iran*. *The emergence and spread of MDR-MRSA strains are considered an ever-growing concern in this region (28). Our research indicated that erythromycin, clindamycin, and ciprofloxacin are not recommended drugs for the treatment of MRSA infections, which is in agreement with the findings reported by other investigators in Iran (25, 29). Antimicrobial resistance was significantly higher among biofilm-producing *S. aureus*. In addition, biofilm is a perfect medium for the exchange of resistance elements. The capability to generate biofilm has been commonly detected in MDR strains, indicating that MDR isolates were more able to produce biofilms compared to the other isolates. Previously, it has been reported that there is a noticeable link between the formation of biofilm and the development of resistant phenotype to gentamicin, erythromycin, clindamycin, cotrimoxazole, and ciprofloxacin among MRSA isolates (30). However, MRSA strains have been demonstrated that exhibited a high level of resistance to other antibiotic classes in a significant portion by various mechanisms, thus, it is difficult to prove the independent effects of biofilm (31). All of the isolates, including MRSA and MSSA, in our study were susceptible to linezolid and vancomycin and the MIC range in MSSA isolate was lower compared with MRSA. This finding is almost in agreement with other reports in Iran. Therefore, these antibiotics can still be administrated for treating drug-resistant *S. aureus* infections (32, 33).

Our analysis indicated that more than 67% of strong and moderate biofilm producers were isolated from the sputum of pediatric patients. According to the previous studies, *S. aureus* isolates from patients with chronic and device-related infection could produce biofilm with more probability than those collected from asymptomatic nasal carriers and other infections (34, 35). Additionally, bacterial biofilm can spread in a layer of mucus or host matrix material during a pulmonary infection (36).

The *icaADBC* genes have been demonstrated to be correlated with biofilm production in *S. aureus*. In the current study, almost all strains, including biofilm and non-biofilm formers, were positive for *icaA*, *icaB*, *icaC*, and *icaD* genes, which is in accordance with the findings reported by another study (37)*.* However, on this point our results differ from other studies (9, 30, 38). Overall, the prevalence of the *icaADBC* genes varies greatly among different investigations. In light of these findings, it can be assumed that elements other than *icaADBC* operon may take part in the formation of biofilm. Additionally, it has been documented that the expression of *icaADBC* is modulated by several genes such as *agr*, *sar*, and *sigB *(39). These regulators probably through interaction with each other may regulate biofilm formation. 

The relationship between methicillin susceptibility and mechanisms of biofilm production in *S. aureus* isolates has been previously documented (40, 41). Accordingly, although biofilm production in clinical MSSA strains, principally, is mediated through an *icaADBC* operon- and PIA production-dependent manner, this generation in MRSA strains is regulated irrespective of *icaADBC *products. In agreement, our results suggested a correlation between the existence of *mecA *and biofilm generation. In the current study, a noticeable association was found between methicillin susceptibility and biofilm-forming, in which MRSA isolates produced more biofilm than MSSA isolates. However, the relationship between the biofilm-forming ability and methicillin susceptibility remains entirely unknown. On the other hand, the studies conducted to characterize biofilm formation among clinical MRSA isolates are limited and controversial. In agreement with our results, several studies have claimed that the production of biofilm and the expression of mobile genetic elements, in particular SCCmec[25] elements, may participate in the antimicrobial resistance (37). It was reported that *SCCmec* type III can be considered a genetic element for the strong biofilm producers (42). Furthermore, we evaluated the presence of MSCRAMM genes, which not only have a role in the adhesion of bacteria to the host matrix, but also participate as a mediator in the accumulation phase of biofilm formation (43, 44). *clfA*, *clfB*, and *fnbpA *genes were extensively distributed among *S. aureus* strains and have no role in the generation of biofilm; however, *fnbpB* was identified in about half of isolates and this gene was more prevalent in MRSA isolates than MSSA. Similar findings have been reported in some previous investigations (45). However, in contrast to our findings on the distribution of these genes, it has been reported that the existence of *clfA* and *fnbpA* in biofilm-forming strains is remarkably greater than in non-biofilm forming *S. aureus* isolates (30, 46). There are some possible explanations. The first concept is that the prevalence of MSCRAMMs profiles is more associated with specific clonal complexes of *S. aureus *(47, 48). Second, the alternation in expression of MSCRAMMs genes can influence biofilm formation, not the presence of them (49). Regarding the *bap* gene, our results emphasized the conclusions of other researchers indicating the absence of the gene in any *S. aureus* isolate of human origin. The *Bap* gene has only been found in a small group of *S. aureus* isolates from bovine mastitis, thus far. Additionally, there is no evidence revealing that the *bap* gene could transfer horizontally in *S. aureus* strains (37, 50). 

## Conclusion

Considering the present study, we found that the production of biofilm and the existence of *fnbB* gene remarkably differ among MRSA and MSSA strains. However, our findings did not indicate the significant role of *icaADBC *genes as major markers for biofilm formation among *S. aureus* isolates. Overall, there is little evidence of specific genetic markers usually attributed to biofilm formation in *S. aureus* isolates from humans and there may be differences in their level of gene expression in MRSA and MSSA isolates. Given the fact that biofilm-producing MRSA infections could induce severe nosocomial infections, there is a necessity to evaluate the presence of MRSA clones in the hospital environment. The application of effective and precise epidemiological typing methods, particularly pulsed-field gel electrophoresis (PFGE) and spa typing, could be beneficial for the management of biofilm producer bacteria in the health care system and patients with susceptible conditions.
